# Genome-wide transcriptome analysis reveals equine embryonic stem cell-derived tenocytes resemble fetal, not adult tenocytes

**DOI:** 10.1186/s13287-020-01692-w

**Published:** 2020-05-19

**Authors:** Y. Z. Paterson, A. Cribbs, M. Espenel, E. J. Smith, F. M. D. Henson, D. J. Guest

**Affiliations:** 1 Department of Veterinary Medicine, University of Cambridge, Madingley Road, Cambridge, CB3 0ES UK; 2 Centre for Preventive Medicine, Animal Health Trust, Lanwades Park, Kentford, Newmarket, Suffolk, CB8 7UU UK; 3 Nuffield Department of Orthopaedics, Rheumatology and Musculoskeletal Sciences, University of Oxford, Oxford, OX3 7LD UK

**Keywords:** Embryonic stem cells (ESCs), Fetal tenocytes, Scar tissue, Transcriptome analysis, Tendon regeneration

## Abstract

**Background:**

Tendon injuries occur frequently in human and equine athletes. Treatment options are limited, and the prognosis is often poor with functionally deficient scar tissue resulting. Fetal tendon injuries in contrast are capable of healing without forming scar tissue. Embryonic stem cells (ESCs) may provide a potential cellular therapeutic to improve adult tendon regeneration; however, whether they can mimic the properties of fetal tenocytes is unknown. To this end, understanding the unique expression profile of normal adult and fetal tenocytes is crucial to allow validation of ESC-derived tenocytes as a cellular therapeutic.

**Methods:**

Equine adult, fetal and ESC-derived tenocytes were cultured in a three-dimensional environment, with histological, morphological and transcriptomic differences compared. Additionally, the effects on gene expression of culturing adult and fetal tenocytes in either conventional two-dimensional monolayer culture or three-dimensional culture were compared using RNA sequencing.

**Results:**

No qualitative differences in three-dimensional tendon constructs generated from adult, fetal and ESCs were found using histological and morphological analysis. However, genome-wide transcriptomic analysis using RNA sequencing revealed that ESC-derived tenocytes’ transcriptomic profile more closely resembled fetal tenocytes as opposed to adult tenocytes. Furthermore, this study adds to the growing evidence that monolayer cultured cells’ gene expression profiles converge, with adult and fetal tenocytes having only 10 significantly different genes when cultured in this manner. In contrast, when adult and fetal tenocytes were cultured in 3D, large distinctions in gene expression between these two developmental stages were found, with 542 genes being differentially expressed.

**Conclusion:**

The information provided in this study makes a significant contribution to the investigation into the differences between adult reparative and fetal regenerative cells and supports the concept of using ESC-derived tenocytes as a cellular therapy. Comparing two- and three-dimensional culture also indicates three-dimensional culture as being a more physiologically relevant culture system for determining transcriptomic difference between the same cell types from different developmental stages.

## Background

Exercise-induced tendon injuries occur commonly and account for 30–50% of all sporting injuries [1]. The Achilles tendon (AT) is particularly vulnerable to injury, along with knee and rotator cuff tendons [2]. AT injuries in humans have remarkable similarities with superficial digital flexor tendon (SDFT) injuries in horses [2, 3]. Both the AT and SDFT serve not only to connect skeletal muscle to bone, but also provide a means of energy-storing to facilitate high-speed locomotion, a function for which no other animal model possesses [3]. Given the less demanding regulatory framework for biological treatments in animals and relative ease of access to equine tissue, the horse is one of the most scientifically sound animal models for studying such injuries [3].

Healing in both human and equine tendon is often prolonged, with injuries undergoing poor natural regeneration and instead healing via fibrotic scar tissue formation. This fibrosis pre-disposes the tissue to high re-injury rates of up to 67% in racehorses and 31% in human athletes [3, 4]. Fetal tendon injuries on the other hand are capable of regenerating without scar tissue formation [5] in a process which is intrinsic to the fetal tendon cells themselves, as fetal tendons transplanted into an adult environment retain their regenerative capacity [6]. This fetal regenerative response has also been observed in other tissues and may be controlled at the cellular level [7–13].

The remarkable healing capacity of fetal cells has driven investigations into their use as a cellular therapy. Fetal skin and tendon progenitor cells are already being tested in clinical trials [14–17]. However, access to human fetal tissue is an issue when considering the use of such treatments in clinical practice. Requirements for parental consent and recent changes in the US federal government’s policies on using human fetal tissue from elective abortions [18] means that sources of fetal tissue are limited. Furthermore, in order to obtain enough cells for transplantation, cells must be serially passaged on tissue culture plastic, which can lead to an altered cellular phenotype due to in vitro selective pressures [19–23]. Regenerative medicine methodologies which mimic fetal-like regeneration are therefore required.

Adult-derived mesenchymal stromal cells (MSCs) are being used in the veterinary field [24]. To date, various MSC sources have been investigated, with companies now offering “off the shelf” MSCs to be used allogeneically in horses [25, 26]. Early phase clinical trials have also been conducted in humans using autologous MSCs for treating rotator cuff disease [27]. However, questions regarding their efficacy remain, and the concept of how MSCs function has changed considerably over the years [28]. MSCs were initially thought to migrate to the injury site, differentiate into functional cells and engraft into the injured tissue. However, the survival rate of injected MSCs is poor [29–31] and it is now hypothesised that MSCs may instead work via modulation of the inflammatory environment [32, 33].

Embryonic stem cells (ESCs) present another potential therapeutic option as they can differentiate into derivatives of all three germ layers, be propagated extensively in culture with manual passaging whilst maintaining a stable karyotype [34–36] and have a degree of immune privilege [32, 37–39]. We have previously isolated and characterised equine ESCs and demonstrated that when injected into the injured horse tendon, they exhibit a high survival rate, express tendon-associated markers and do not illicit an immune response within a 90-day study period [29, 40–42]. However, the long-term survival/rejection of these cells is unknown, and studies in human and mouse models have indicated a high likelihood of teratoma formation based on injection into other transplantation sites [43]. To avoid this risk, strategies to induce human and mouse ESCs towards the appropriate lineage prior to clinical application have been investigated [44–49].

Equine ESCs differentiate into tenocytes in vitro in response to transforming growth factor-β3 (TGF-β3) and three-dimensional (3D) culture [42, 50, 51]. Similarly, human ESCs have been differentiated into tenocytes in vitro [52, 53]. Transplantation of differentiated ESC-derived 3D fibrin tendon gels into rat patella tendon injury models has indicated that ESC-tenocytes secrete a number of fetal tendon matrix and differentiation factors [52], highlighting the potential role ESC-tenocytes may play in tissue regeneration. However, to date, a full transcriptional characterisation of ESC differentiated tenocyte progeny has not been performed. In this study, next-generation sequencing was performed using equine fetal, adult and ESC-tenocytes cultured in 3D allowing for comparison of their transcriptomic profiles. 3D culture was also compared to its typical two-dimensional (2D) counterpart to highlight the detrimental effects of culturing cells in conventional monolayer systems.

## Materials and methods

### Tenocyte cell culture

Tendons were processed with the approval of the Animal Health Trust ethical review committee (AHT02_2012) as previously described [50]. Tenocytes were isolated post-mortem from healthy SDFTs of eight adult Thoroughbred horses (2–10 years) that were euthanised for reasons unrelated to this project, and from tendons of seven fetuses at 271, 289, 316, 319, 320, 321 and 340 days of gestation which had undergone spontaneous abortion. Briefly, dissected tissue was digested overnight at 37 °C in 1 mg/ml collagenase (Sigma, Poole, Dorset, UK) solution. Cells were expanded in Dulbecco’s modified Eagle’s Medium (DMEM) (Gibco, Invitrogen, Carlsbad, CA, USA) with 10% fetal bovine serum (FBS) (Gibco), 1% penicillin-streptomycin (P/S) (Gibco) and 2 mM l-glutamine (LQ) (Gibco). Culture conditions were maintained at 37 °C in 5% CO_2_, with medium being replaced every 2–3 days. Once cells reached 80% confluency, they were passaged using 0.25% trypsin/EDTA (Sigma).

### Embryonic stem cell culture

Three lines of previously characterised ESCs [40, 41], isolated from three different embryos, were used. ESCs were maintained at 37 °C in 5% CO_2_ on a mitotically inactivated mouse embryonic fibroblast layer as previously described [40, 41]. Briefly, cells were cultured in DMEM/F12 containing 15% FBS (Gibco), 2 mM LQ, 1% non-essential amino acids, 1 mM sodium pyruvate, 0.1 mM 2-mercaptoethanol (all from Invitrogen, Renfrewshire, UK) and 1000 U/ml leukaemia inhibitory factor (LIF) (Cambridge University, Biochemistry Department, UK). ESCs were passaged mechanically every 5–7 days in the presence of 2 μM Thiazovivin (StemGent, Cambridge, MA, USA), with medium replaced daily.

### Three-dimensional cell culture and ESC differentiation

3D cell culture was conducted as previously described [50, 54] on silicon-coated six-well plates (Sylgard 184 Silicone elastomer; Dow Corning) with pairs of 0.2-mm-diameter minutien pins (Interfocus fine science tools) embedded at 15 mm distances. Tenocytes were suspended (4 × 10^5^ cells/ml) in a chilled mixture of eight parts PureCol (Bovine collagen type I; Advanced Biomatrix, Carlsbad, CA, USA) to 2 parts medium (pH adjusted from 7.2 to 7.6 with 1 M sodium hydroxide), with 200 μl being pipetted around each pair of pins before allowing to set at 37 °C for 60–90 min. Once set, 3 ml of tenocyte medium was added to each well. For tendon differentiation, mechanically passaged clumps of ESCs were used to prepare three-dimensional constructs at the same cell density and were cultured in ESC medium without LIF, a protocol which our group has shown is sufficient to promote tenogenic differentiation in equine ESCs [50]. 3D cultures were maintained for 14 days with the medium changed every 3–4 days. ImageJ software (National Institutes of Health) was used for contraction analysis with values displayed as a percentage of the day 0 value.

### RNA isolation

Three lines of fetal, adult and ESC-tenocytes cultured in 3D and adult and fetal tenocytes cultured in 2D were used in the RNA-sequencing experiments. The 2D samples, although processed in parallel, are available through NCBI GEO, (https://www.ncbi.nlm.nih.gov/geo/) under accession number GSE132358 [39]. Tenocytes were used between passages (P) 3 and P8. Differentiated ESCs were used between P12 and P22. Quantitative PCR (qPCR) validation of the RNA-sequencing data was conducted using eight lines of adult tenocytes and seven lines of fetal tenocytes. RNA was harvested in 1 ml Tri-reagent (Sigma) per six to nine gel constructs for 3D-cultured cells or per 10 cm^2^ plate for 2D-cultured cells. RNA was extracted and purified using a RNeasy mini kit (Qiagen, Manchester, UK). The Ambion DNA-free kit was used to remove any contaminating DNA (Life Technologies, Paisley, UK). RNA concentration was measured using a Nanodrop (ThermoFisher, Loughborough, UK). RNA integrity was confirmed on an Agilent 2200 Tapestation by an external provider (Cambridge Genomics, Cambridge, UK), with RNA Integrity Number (RIN/RINe) values of 9.1–10 obtained.

### RNA sequencing

mRNA library prep and sequencing was performed by external providers (Edinburgh Genomics, Edinburgh, UK and Otogenetics, Atlanta, USA) using a TruSeq stranded mRNA kit (Illumina, Cambridge, UK) or Clonetech Smart cDNA kit (Clonetech laboratories, CA, USA) and the NovaSeq6000 (Illumina) and Illumina HiSeq2500, respectively. Twenty million reads of approximately 100 base pair paired-end were generated per sample. FASTQC and FASTQ Screen (Babraham Bioinformatics, Cambridge, UK) were used on the FASTQ files to ensure quality. Reads were aligned to the Ensemble version v96 EquCab 3.0 transcriptome using the pseudoaligner Salmon [55] with GC-bias correction (-gcBias) in Quasi-mapping based mode. Tximport [56] was used to import the gene-level abundance data into R (v.3.5.2) and R/Bioconductor DSeq2 (v.1.22.2) used to conduct differential expression analysis as described in Love et al. [57], with batch correction applied. Genes with Log2FC of ± 2 and adjusted *p* value (p-adj) of < 0.01 were considered differentially expressed (DE). Gene Ontology (GO) analysis was performed using Panther (http://www.pantherdb.org/), with a false discovery rate (FDR) of < 0.05 being defined as significantly enriched. Pathway analysis was performed using Gene Analytics from the LifeMap’s GeneCards Suite (http://geneanalytics.genecards.org) with entity scores of > 13 being classed as highly enriched.

### cDNA synthesis and quantitative PCR

One microgram of RNA was reverse transcribed using the SensiFAST cDNA Synthesis Kit (Bioline, London, UK). Reactions lacking the reverse transcriptase were carried out to ensure no genomic DNA contamination. Equine gene-specific primers were designed using NCBI Primer-Blast (https://www.ncbi.nlm.nih.gov/tools/primer-blast/) (Additional file 1: Table S1). qPCR was carried out on the Bio-Rad C1000 Touch Thermal Cycler (Bio-Rad), using supermix containing SYBR Green (Bioline). qPCR cycling parameters were 95 °C for 10 min, followed by 45 cycles of 95 °C for 15 s, 60 °C for 15 s and 72 °C for 15 s. Reactions were quantified relative to 18s rRNA housekeeping gene [58], whose stability was determined using RefFinder [59] (data not shown). The relative gene expression was calculated using: 1/2^ΔΔCT^ [60]. Statistical significance in mean gene expression was determined using a Student’s *t* test.

### Serial passaging effects

Three lines of adult tenocytes were isolated and passaged ten times in 2D culture, with RNA extracted at every passage to measure *COL1A2*, *SCX*, *THBS4* and *TNMD* gene expression via qPCR. Statistical significance was tested using linear regression analysis using R (v.3.5.2).

### Histology and immunocytochemistry on sectioned 3D constructs

Three lines of adult, fetal and ESC-tenocyte 3D constructs were embedded in OCT compound (VWR) and snap-frozen in liquid nitrogen-cooled isopentane. Eleven-micrometre-thick, longitudinal sections were cut using a cryostat, fixed for 10 min in 100% acetone and stored at − 20 °C. Sections were used for haematoxylin and eosin (H&E) and Picro Sirius Red staining (ab150681, Abcam, Cambridge, UK).

For immunocytochemistry, sections were blocked in 2.5% normal horse serum (Vector Laboratories, Peterborough, UK) for 20 min before incubating with primary antibodies overnight at 4 °C, with subsequent detection using fluorescently labelled secondary antibody. Antibodies were used at optimised concentrations in 2.5% normal horse serum (Vector Laboratories) (Additional file 1: Table S2). Colorimetric images were captured using the Olympus IMT2-SFR inverted microscope, with polarising filters added for visualising Picro Sirius Red staining (Abcam). Fluorescence images were captured using the Zeiss Axioplan 2 imaging suite. Subsequent images were analysed qualitatively.

### Immunocytochemistry on 2D coverslips

Three lines of adult and fetal tenocytes were cultured on gelatin-coated (Sigma) coverslips in 1% serum medium and 24 h later incubated with or without the addition of 5 ng/ul TGFβ-3 (Peprotech, London, UK) for 30 min before fixing for 20 min in 3% paraformaldehyde. Cells were permeabilised in 0.1% triton-X-100 for 1 h. Primary and secondary antibody incubations were done as above for 3D-cultured cells (Additional file 1: Table S2).

### Migration assay

Four biological lines of adult and fetal tenocytes were treated with 10 μg/ml of mitomycin C (Sigma) at 37 °C for 2 h to inhibit cell proliferation prior to culture in 1% serum medium. Following mitomycin C treatment, 3.82 × 10^4^ cells were plated into each well of a 24-well Transwell® plate, pore diameter 8 μm (Corning Incorporated, NY, USA). Growth factors (5 ng/ul TGFβ-3, 10 ng/ml bFGF, 5 ng/ml PDGF, 10 ng/ml and 5 ng/ml, respectively, bFGF + PDGF, 100 ng/ml IGF-1 (Peprotech)) were added to the lower well for 24 h of culture. The inserts were fixed for 20 min at room temperature in 3% paraformaldehyde and washed 3 times in PBS and the cells on the upper membrane were removed with a cell scraper (ThermoFisher). The remaining cells (on the lower side of the membrane) were stained for 10 min in 1% crystal violet solution (Sigma), and five images at × 4 magnification and nine images at × 10 magnification were taken on an EVOS XL Core Cell Imaging System (ThermoFisher). Image analysis was performed using ImageJ software with the average cell coverage being calculated and statistically significant effects being determined via a Student’s *t* test.

## Results

### Serial passaging of equine tendon cell alters gene expression

The effect of serial passaging on the expression of four tendon-associated genes, *COL1A2*, *SCX*, *THBS4* and *TNMD*, in equine tenocytes was measured in this study. *COL1A2* expression was variable with passaging, with a significant difference from P0 only being observed at P6 and P7 (*p* value = 0.0256 and 0.0027, respectively) (Additional file 2: Figure S1A). *SCX* expression at P0 was significantly higher than from P4 through to P10 (*p* values all < 0.05) (Additional file 2: Figure S1B). However, *SCX* levels did not change beyond P4 with further passaging. *THBS4* at P0 was significantly higher than from P1 through to P10 (*p* values all < 0.05) (Additional file 2: Figure S1C). However, *THBS4* levels did not change beyond P4 with further passaging. *TNMD* at P0 was significantly higher than at P1 through to P10 (*p* value = 0.009). However, TNMD expression did not change beyond P1 with further passaging (Additional file 2: Figure S1D).

### Cellular characterisation of adult, fetal and embryonic tenocytes cultured in 3D reveals no quantitative or qualitative differences

**Fig. 1:**
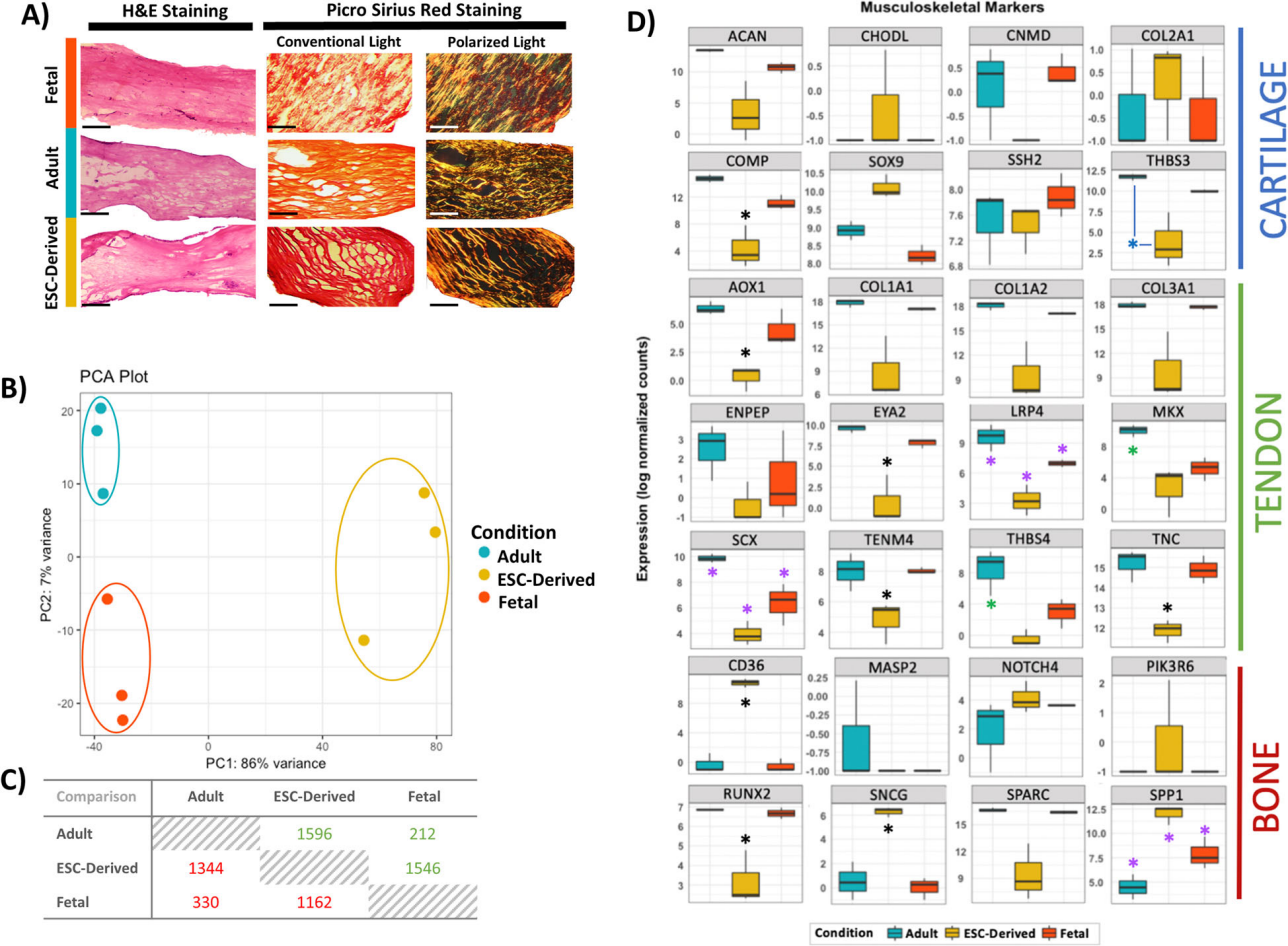
Morphological and transcriptomic comparison of adult, fetal and ESC-tenocytes. **a** Haematoxylin and eosin (H&E) and Picro Sirius Red staining of adult, fetal and ESC-derived 3D constructs. All cell types show similar collagen fibre alignment and collagen content within the constructs after 14 days of culture. Scale bar for H&E = 0.5 mm. Scale bar for Picro Sirius Red = 250 μm. Images shown are representative of three biological repeats. Under light microscopy, all collagen fibres are red following Picro Sirius Red staining, under polarised microscopy collagen type I fibres have yellow-orange birefringence and collagen type III fibres have green birefringence. **b** Principle component analysis of gene expression data profiling three biological lines of 3D cultured adult, fetal and ESC-tenocytes. **c** Matrix of up (green) and down (red) regulated genes for each of the pairwise comparisons. Values represent the number of up- and downregulated genes based on an adjusted *p* value of < 0.01 and a Log2FC of ± 2. **d** Boxplots of gene expression of cartilage, tendon and bone markers in adult (teal), fetal (orange) and ESC-tenocytes (yellow). *Y*-axis shows the gene expression in terms of the log normalised counts. Significant differences are depicted by an asterisk (*) with black asterisks representing a significant difference between the ESC-tenocytes and both the adult and fetal tenocytes, blue asterisks representing a significant difference between the ESC-tenocytes and adult tenocytes only, green asterisks representing a significant difference between the adult tenocytes and both the ESC and fetal tenocytes and purple asterisks representing a significant difference between all three groups

We have previously demonstrated that equine ESCs can differentiate into tenocytes in both 2D and 3D culture, showing increased differentiation in 3D [50]. 3D constructs cultured for 14 days showed no significant differences in the degree of contraction between cell types (data not shown). Collagen fibre alignment or cell distribution between cell types was also not qualitatively different (Fig. 1a and Additional file 2: Figure S2). Picro Sirius Red staining similarly indicated that the relative staining of type I collagen to type III collagen was not qualitatively different between cell types (Fig. 1a and Additional file 2: Figure S2).

### Embryonic tenocytes cultured in 3D are transcriptomically more similar to fetal than adult tenocytes but represent a unique population

Gene expression profiles obtained by RNA sequencing of the entire equine genome yielded 6190 DE genes in total across the three 3D-cultured cell type comparisons (Log2FC of ± 2 and p-adj of < 0.01) (Fig. 1c). Principle component analysis (PCA) showed clear segregation of the three cell types, with the greatest variance on PC1 separating the ESC-tenocytes from the adult and fetal tenocytes (Fig. 1b). Of the 21,689 mapped genes, 542 genes were DE between the adult and fetal tenocytes, 2940 genes were DE between the adult and ESC-tenocytes and 2708 genes were DE between the fetal and the ESC-tenocytes. Gene-level summaries can be found in Additional file 3.

To further identify the cells as being tenocytes, a panel of 28 musculoskeletal-associated genes identified from the current literature [61–65] were examined. ESC-tenocytes expressed all tendon-associated genes, however, at lower levels compared to adult and fetal tenocytes (Fig. 1d). Of the eight cartilage genes, only *COMP* and *THBS3* showed significant differences between the cell types, with both markers having the lowest expression in ESC-tenocytes. The bone genes *CD36*, *SNCG* and *SPP1* were expressed significantly more in the ESC-tenocytes, but *RUNX2* was expressed at a significantly lower level in the ESC-tenocytes.

**Fig. 2:**
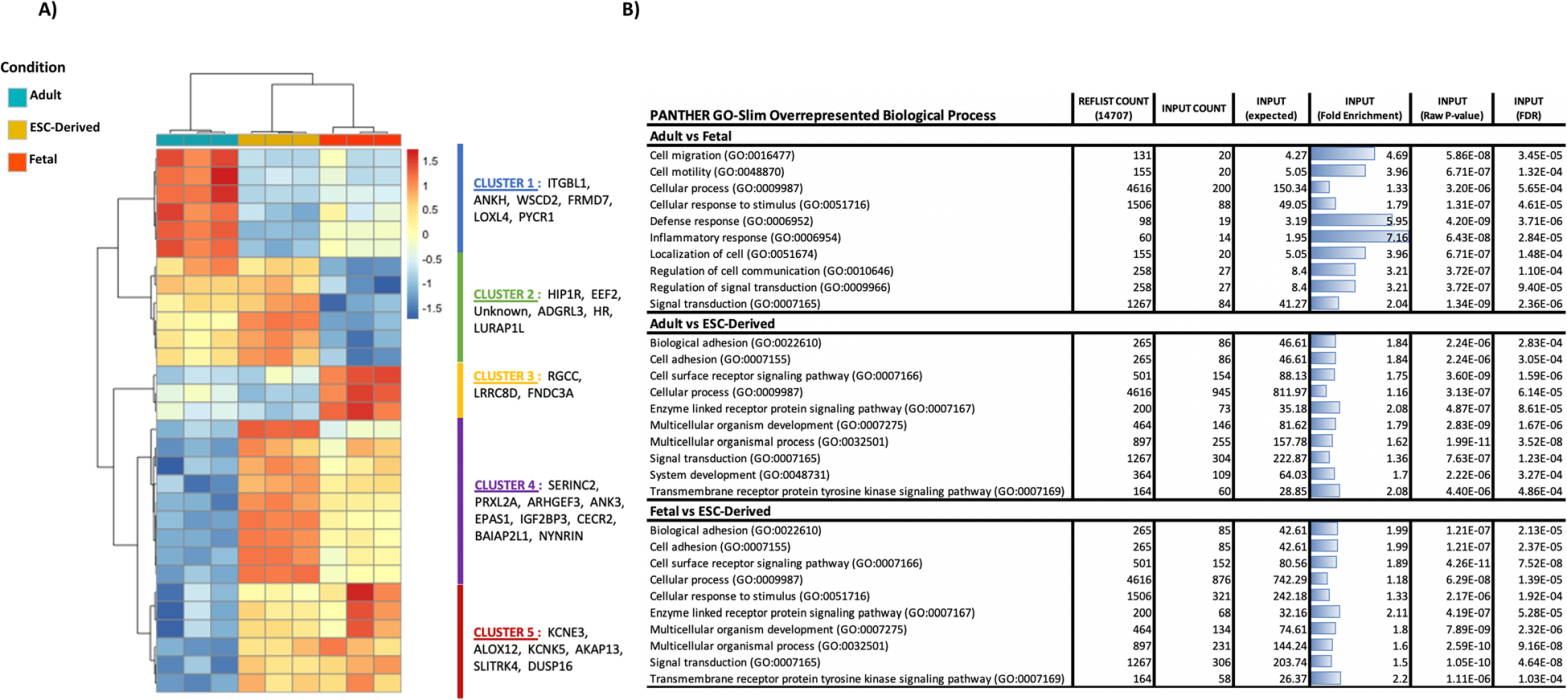
Heatmap of top DE genes and GO terms comparing adult, fetal and ESC-tenocytes. **a** Differential gene expression analysis for top 30 genes DE in adult and fetal tenocytes. Dendrograms are based on Pearson’s correlation, with red and blue colours representing up- and downregulated genes, respectively. Samples with intermediate expression are represented in yellow. Groups are visualised in columns, with the coloured bar above the heatmap indicating the grouping variable. Genes are represented in individual rows, with patterns of up- and downregulated genes being classified into 5 clusters. **b** Summary of the top 10 significantly enriched gene ontology (GO) biological process terms for each pairwise comparison of DE genes in adult, fetal and ESC-derived tenocytes. Go terms are arranged alphabetically to allow easier comparison between each group

To better understand the differences between fetal and adult tenocytes, a heatmap was constructed of the top 30 DE genes. This resulted in dendrogram clustering of the fetal tenocytes with the ESC-tenocytes, with 21 out of the 30 genes showing a more similar expression pattern than with adult tenocytes (Fig. 2a). Cluster 1 highlights six genes which are highly upregulated in adult tenocytes, whereas clusters 4 and 5 show 15 genes which are highly downregulated in adult tenocytes. Two clusters, 2 and 3, consisting of 9 genes in total, show a more closely related expression between ESC-tenocytes and adult tenocytes. Heatmaps of the top 100, 200 and 500 genes were also constructed and resulted in similar dendrogram clustering of the fetal tenocytes with the ESC-tenocytes (Additional file 2: Figure S3).

### Comparison of overrepresented gene ontologies and pathways in adult, fetal and embryonic tenocytes cultured in 3D

Gene ontology (GO) analysis was performed on the genes identified as being DE between the three different groups. The top 10 over-enriched terms are listed in Fig. 2b. Next to the more common terms (e.g. regulation of cell communication, signal transduction, and cellular processes), DE genes between adult and fetal tenocytes were assigned to processes involving the inflammatory response, cellular migration and motility. Comparing ESC-tenocytes to adult and fetal tenocytes revealed pathways involved in organism processes, development and biological and cellular adhesions as being significantly overrepresented (Fig. 2b). DE genes were then overlaid into GeneAnalytics Pathway Analysis. ERK, PAK, Akt and PI3K-Akt signalling were differentially regulated among all three groups, as was extracellular matrix (ECM) degradation (Additional file 1: Table S3). In corroboration with the GO analysis, cytokine signalling in the immune system was found to be differentially regulated in adult and fetal tenocytes (Additional file 1: Table S3).

### Validation of 3D RNA-seq results by qPCR and immunofluorescence is highly corroborative

**Fig. 3:**
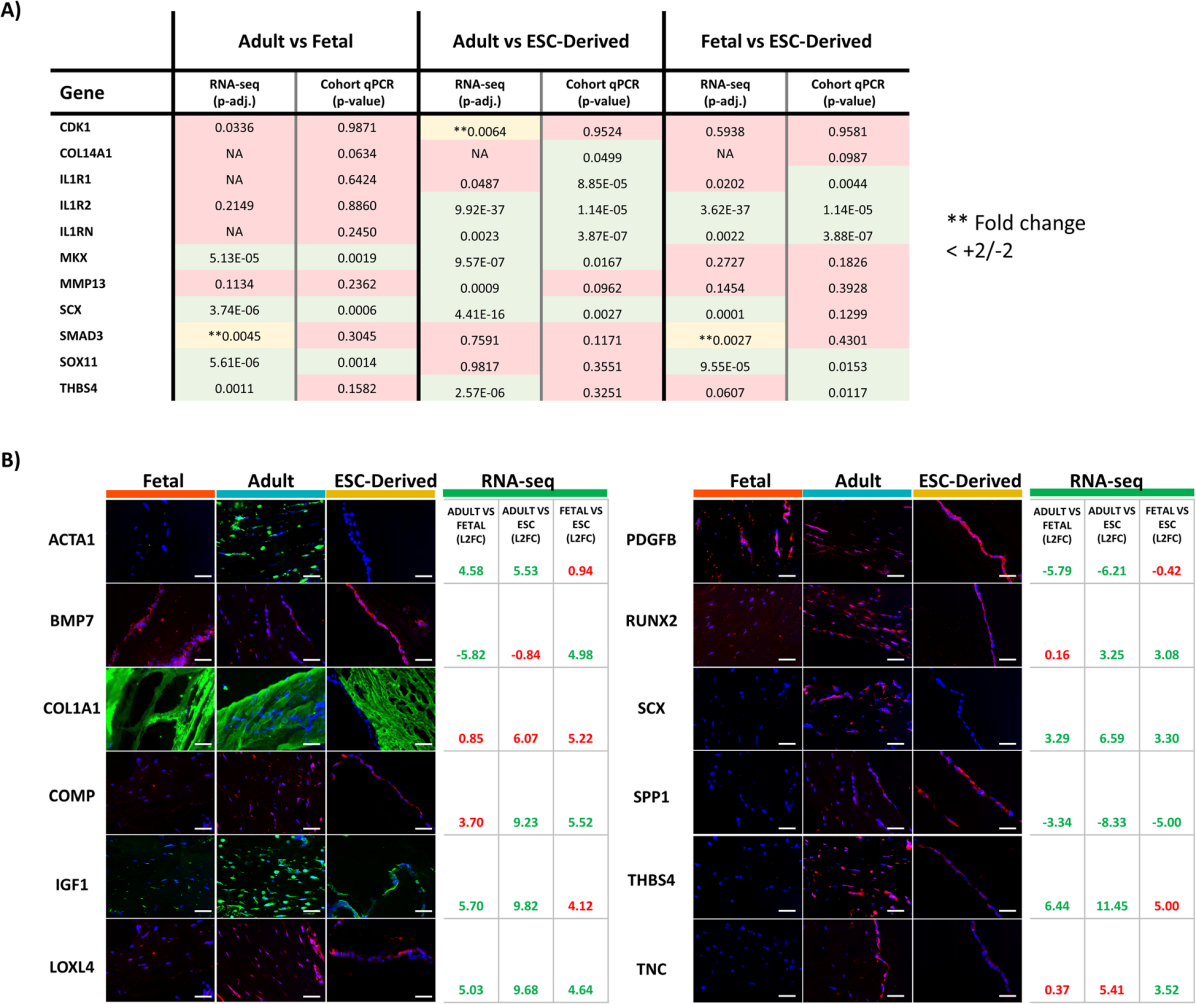
Validation of RNA-sequencing results. **a** Comparison of DE genes using RNA-seq and qPCR results using a log2FC cut-off ± 2. Significance threshold for RNA-seq p-adj values < 0.01 as routinely used. Significance threshold for qPCR *p*-value < 0.05 as routinely used. Red shaded boxes indicate no significant difference. Yellow shaded boxes represent a significant p-adj value; however, they do not meet the log2FC cut-off. Green shaded boxes indicate there is a significant difference based on both p-adj/*p* value and log2FC. NA values present typically arise due to count outliers as detected by Cook’s distance. **b** Immunocytochemistry comparison of the corresponding proteins for 12 significantly DE genes. Images represent longitudinal sections of 3D adult, fetal and ESC seeded constructs following 14 days of culture (*n* = 3 for each cell type). RNA-sequencing log2FC are listed in the adjoining tables with green text indicating significance based on p-adj value of < 0.01 and red text indicating no significant difference. Scale bar = 40 μm. DAPI staining of the nuclei is shown in blue

To validate the biological significance of the RNA-seq data, several DE genes were investigated at either the RNA level, protein level or both (Fig. 3a, b). For qPCR validation, the original samples sequenced were used alongside an additional cohort of adult (total *n* = 8) and fetal tenocytes (total *n* = 7). Of the eleven genes tested, there was a 79% corroboration to that of the RNA-seq data (Fig. 3a). Validation via immunocytochemistry, although not quantitative, showed a high consensus to that of the sequencing data, with ACTA1, IGF1, LOXL4, SCX and THBS4 having visibly higher protein expression in the adult tenocytes compared to the fetal and ESC-tenocytes as predicted (Fig. 3b). Protein expression of PDGFB and RUNX2 did not corroborate with sequencing data, and no visible differences were observed between the three groups (Fig. 3b). BMP7 expression was expected to be absent from adult and ESC-tenocytes based on the RNA-seq data; however, protein was detectable in the ESC-tenocytes and adult tenocytes, although at a lower level (Fig. 3b).

### Adult and fetal tenocytes respond differently in their migratory abilities upon addition of growth factors

**Fig. 4:**
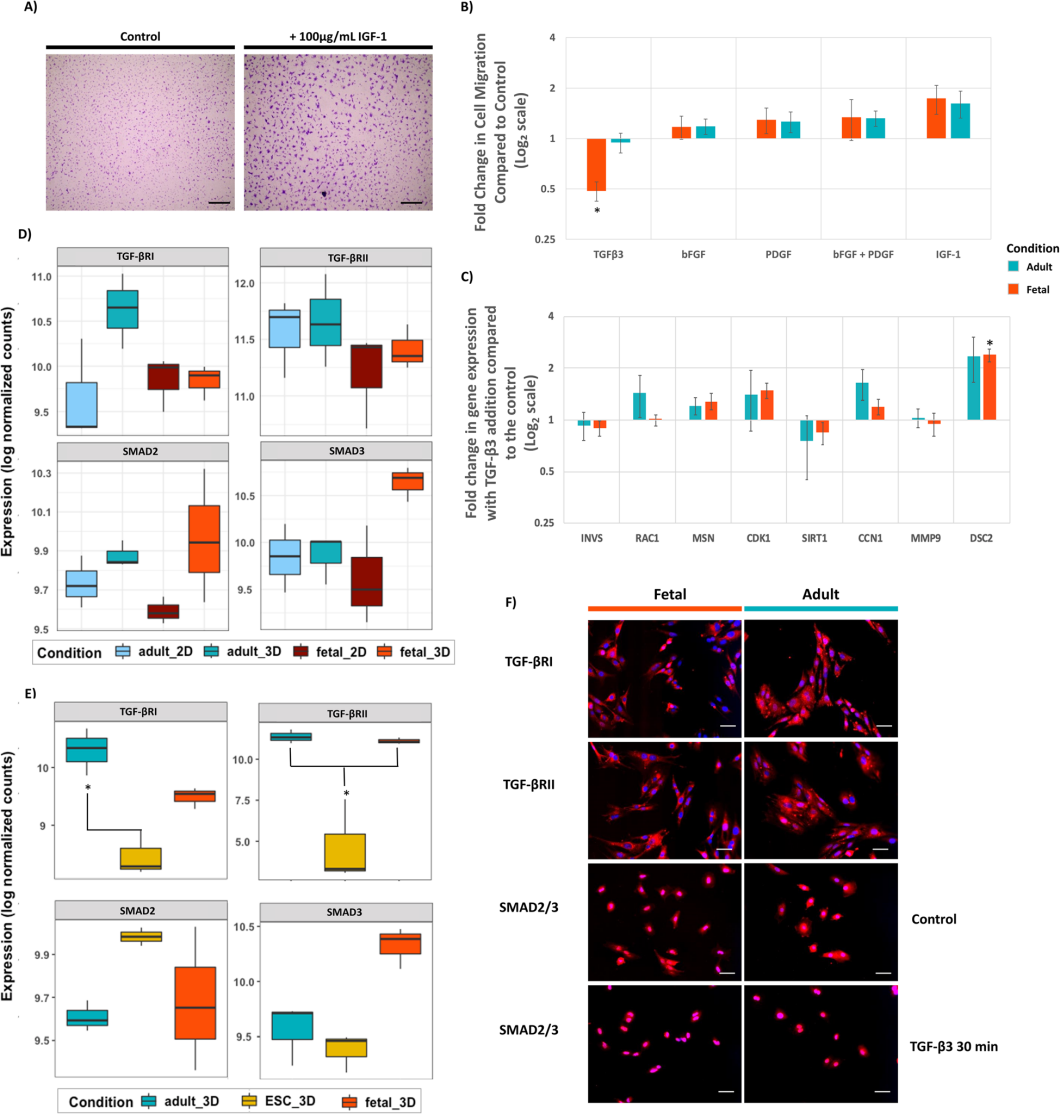
Comparison of migratory properties in response to growth factors. **a** Representative image of crystal violet staining of adult tenocytes with and without the addition of 100 ng/ml IGF-1 grown in 1% serum tenocyte medium. Scale bar = 500 μm. Images shown are representative of three biological repeats of both adult and fetal tenocytes. **b** Log2FC in cell migration in adult and fetal tenocytes following 24-h exposure to growth factors compared to the control. Error bars represent the s.e.m. of three biological replicates. *p < 0.05 based on Student’s *t* test. **c** Log2FC in gene expression in adult and fetal tenocytes following exposure to TGF**-**β3 compared to the non-exposed control. Error bars represent the s.e.m. of three biological replicates. Student’s *t* test *p < 0.05. **d** Boxplots showing the log normalised expression counts of TGF-β receptors 1 and 2 and *SMAD2* and *SMAD3* in adult and fetal tenocytes cultured in both 2D and 3D. Statistical significance based on an adjusted *p* value of < 0.01 and a Log2FC of ± 2 (*). **e** Boxplots showing the log normalised expression counts of TGF-β receptors 1 and 2 and *SMAD2* and *SMAD3* in adult, fetal and ESC-tenocytes cultured in 3D. Statistical significance based on an adjusted *p* value of < 0.01 and a Log2FC of ± 2 (*). **f** Immunofluorescence staining of TGF-β receptors 1 and 2 and *SMAD2/3* in adult and fetal tenocytes cultured in 2D. SMAD_2/3_ is translocated to the nucleus of both adult and fetal tenocytes following 30 min of TGF-β3 exposure. DAPI staining of the nucleus is shown in blue. Scale bar = 40 μm. Images shown are representative of three biological repeats

As genes involved in cell migration were DE between adult and fetal tenocytes, cell migration assays were performed. Growth factors bFGF and PDGF either alone or in combination had no significant effect on adult or fetal tenocytes (Fig. 4b). IGF-1 resulted in marked morphological changes (Fig. 4a) and appeared to show an increased migration rate, but this was not significant (*p* value 0.052 and 0.058, respectively, for adult and fetal tenocytes) (Fig. 4b). However, TGF-β3 significantly inhibited cell migration in fetal tenocytes but had no effect on adult tenocytes (*p* values 3.9 × 10^− 6^ and 0.69, respectively) (Fig. 4b). To further explore this difference in migration in response to TGF-β3, qPCR was used to test several genes involved in cellular migration; however, no significant differences were found between the adult and fetal cells (Fig. 4c).

TGF-β receptor expression in fetal, adult and ESC-tenocytes was also compared. TGF-β receptor 1 (*TGF-βRI*) was not DE in adult and fetal tenocytes cultured in 2D or 3D; however, ESC-tenocytes in 3D expressed significantly less *TGF-βRI* than adult tenocytes (Log2FC 2.064, p-adj 3.41e−09) (Fig. 4d, e). Similarly, no differences were observed in *TGF-βR2* expression between adult and fetal tenocytes in 2D or 3D culture; however, ESC-tenocytes in 3D expressed significantly less *TGF-βRII* than both adult tenocytes (Log2FC 5.385, p-adj 6.29e−03) and fetal tenocytes (Log2FC 5.083, p-adj 0.0014) (Fig. 4d, e). As mRNA does not always corroborate with protein expression, as described in Fig. 3b, immunofluorescence staining on 2D-cultured cells was performed to confirm and compare expression of both receptors in adult and fetal tenocytes (Fig. 4f). *SMAD2/3*, which translocates in response to TGF-β3 signalling, has similar gene expression between fetal, adult and ESC-tenocytes cultured in both 2D and 3D (Fig. 4f). Immunofluorescence staining showed clear translocation of SMAD2/3 into the nucleus in the presence of TGF-β3 in both adult and fetal tenocytes (Fig. 4f). As 3D culture does not allow for clear visualisation of cell nuclei, receptor staining and subsequent translocation assays were only performed in 2D-cultured cells.

### 2D monolayer culture results in convergence in the expression profiles of adult and fetal tenocytes

**Fig. 5:**
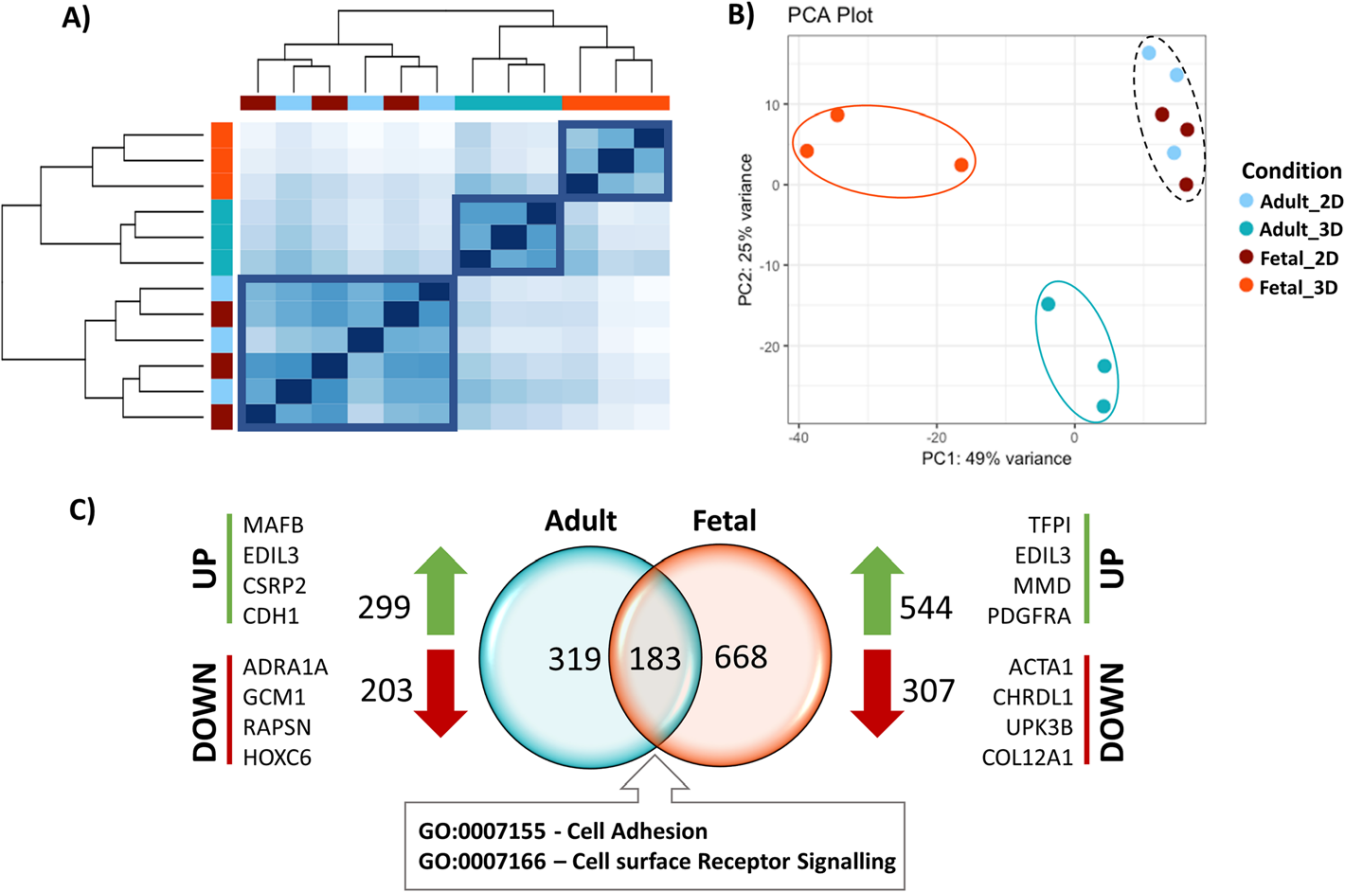
Comparison of gene expression changes upon 2D and 3D culturing of equine tenocytes. Comparison of RNA transcriptome profile between three biological lines of fetal and adult tenocytes cultured in either a 2D or 3D. **a** Euclidean sample-to-sample distance matrix with hierarchical clustering using VST transformed data. **b** Principle component analysis of gene expression data profiling three biological lines of 3D versus 2D cultured adult and fetal tenocytes. **c** Quantitative VENN diagram showing overlap of DE genes in the adult 3D vs 2D culture comparison (teal) and fetal 3D vs 2D culture comparison (orange). Top 4 upregulated genes based on their p-adj value are shown in both groups next to the green arrow and the top 4 downregulated genes value are shown in both groups next to the red arrows

Hierarchical clustering and PCA were conducted on adult and fetal tenocytes cultured in either 2D or 3D (Fig. 5a, b). Analysis revealed a convergence of expression profiles in 2D, with only 10 genes being DE. This is in comparison to 542 DE genes in 3D. When comparing adult tenocytes cultured in either 2D or 3D culture, 502 genes were DE, whereas in fetal tenocytes cultured in either 2D or 3D culture, 851 genes were DE. Of these, only 183 genes were common between adult 2D versus 3D culture and fetal 2D versus 3D culture (Fig. 5c). GO analysis of these 183 genes revealed cell adhesion (GO:0007155) and cell surface receptor signalling (GO:0007166) as key overrepresented biological processes (Additional file 1: Table S4). Degradation of the ECM was a common pathway which was differentially regulated in both adult and fetal tenocytes as a result of 2D or 3D culture, with differences in collagens, matrix metalloproteinases and integrins (Additional file 1: Table S5). Muscle contraction was a predominant pathway which was found to be enriched in monolayer cultures, whereas TGF-β, ERK, PAK and Akt signalling was enriched in 3D-cultured cells.

## Discussion

Therapies utilising pluripotent stem cell derivatives are an expanding market, with around 30 clinical trials currently ongoing [66]. Tenocytes derived from ESCs may too prove a useful cellular therapeutic for promoting scar-free tendon repair, like that observed in fetal tendons. However, the difficulties associated with tenocyte identification and a lack of direct comparative studies on ESC-tenocytes makes it difficult to assess their potential. Here we report for the first time the transcriptional profiling of adult, fetal and ESC-tenocytes cultured in a 3D environment and show that ESC-tenocytes are transcriptomically closer to fetal as opposed to adult tenocytes. We also demonstrate that culturing adult and fetal cells in 3D results in a large divergence of their gene expression profiles in comparison to 2D static monolayer culture. This result indicates that 3D culture is a more robust method to assess differences in the same cell types from different developmental stages, which would otherwise be lost in conventional culture systems. Collectively, these results indicate that 3D-differentiated ESC-tenocytes may be a useful cell source for the treatment of tendon injuries in the future.

This data suggests that our differentiation protocol, like others [42, 50, 67–70], mimics normal tenocyte development. As previously described, the contraction rates and survival of 3D adult, fetal and ESC constructs showed no significant differences, with histological analysis showing similar cellular morphology and alignment of the collagen fibres [50]. Collagen type I and type III, which are the major components in tendon tissue, did not significantly differ in gene expression and no qualitative differences were determined histologically. Additional quantification of the precise collagen content and fibre alignments would be needed to confirm this. Although there are many differences in gene expression between ESC-derived, fetal and adult tenocytes (suggesting all three groups represent unique populations), this study shows that of the 26,991 genes mapped, ESC-tenocytes have fewer DE genes when compared to fetal tenocytes (2708 genes) as opposed to adult tenocytes (2940 genes). Results similar to this have been described in ESC-derived human neural stem cells, where 2041 genes were DE compared to fetal neural stem cells [71], and ESC-derived human pancreatic β cells, where 755 genes were DE compared to adult pancreatic β cells [66].

One of the main challenges in assessing tendon differentiation is the lack of definitive markers to identify tendon populations [72] and a panel of tendon-related genes is usually used. Yet, many of these tendon-related genes are widely expressed in various tissues [13, 72, 73]. Similarly, Zhang et al. highlighted the lack of available micro-array and RNA-seq datasets documenting the transition from fetal to adult tendon, with most datasets only comprising of the initial stages of embryonic limb development [72], therefore what stage of tendon development differentiated progeny represent is not always apparent. This study further highlights the issues in this form of assessment. Of the 12 tendon-related genes commonly investigated, four were found to be DE between adult and fetal tenocytes. Three of these genes *THBS4*, *SCX* and *MKX* [74, 75] are some of the most commonly cited “tendon” genes, and interestingly, all show a clear gradient of expression, with adult tenocytes having the highest expression, followed by the fetal and then ESC-tenocytes. We also demonstrate that *SCX* and *THBS4* are rapidly downregulated in 2D culture and the response of all three of these putative marker genes to 2D and 3D culture differs between adult and fetal cells. They may therefore not be suitable as markers of a regenerative cell type.

Our work does not address the heterogeneity of our sample populations. Although we believe that 3D culture is a potent driver of ESC differentiation into tenocytes, quantification of the percentage of desired cell fate achieved has not been conducted [50]. Yet even in very optimised differentiation protocols in human ESCs, significant variation in differentiation efficiencies is found, with certain lines more readily differentiating down certain lineages than others due to epigenetic, cell cycle patterns and genetic differences between lines [76–78]. To try and counteract this problem, cell sorting is typically performed [79]; however, this relies on a suitable marker being available. Similarly, we have not measured the heterogeneity within the tendon population itself. Studies using single-cell RNA-seq suggest that different sub-populations of tenocytes exist [80], with multiple progenitor populations having been derived [81]; therefore, further study to identify sub-populations of cultured tenocytes is required. The heterogeneous nature of the ESC-tenocyte population is therefore likely, in part, to explain why the musculoskeletal gene expression profile is less robust in ESC-tenocytes compared to both adult and fetal tenocytes.

Wound healing occurs firstly via an acute and local inflammatory response; this leads to cellular proliferation and ECM deposition to remodel the injured tissue [82]. In adults, there is rapid cellular proliferation and a delayed but excessive accumulation of unorganised ECM resulting in scar tissue. In contrast, fetal wounds regenerate with non-disrupted collagen ECM through simultaneous proliferation and synthesis of organised collagen [83]. Although the exact mechanisms of fetal scar-less healing are unknown, various studies suggest this regenerative ability is partly explained by intrinsic differences between adult and fetal fibroblasts themselves. Comparative studies have indicated that differences in the migratory activity; inflammatory responses; cellular-mediated expression of chemokine, cytokines and growth factors; and deposition of components of the ECM may play a vital role [11, 83–85].

One of the top DE genes between adult and fetal tenocytes was *LOXL4*, a member of the lysyl oxidase gene family which are responsible for initiating collagen and elastin crosslinking in the ECM via signalling through the TGF-β pathway [86]. *LOXL4* has been heavily implicated in fibrosis in skin and vascular tissue [87] and is downregulated in Murphy Roth Large (MRL) mice which heal without scarring [88]. Interestingly, this gene is significantly downregulated in both fetal and ESC-tenocytes (− 5.03 and − 9.68 logFC, respectively) compared to adult tenocytes, a result which was confirmed at both the mRNA and protein level. Other genes within this top cluster that are downregulated in both fetal and ESC-tenocytes, compared to adult tenocytes, include the Integrin Subunit Beta Like 1 (*ITGBL1)* gene which can promote chondrogenesis [89] and *ANKH*, an inorganic pyrophosphate transport regulator, which has been linked to calcification in articular cartilage. The relevance of other genes within this cluster, *WSCD2*, *FRMD7* and *PYCR1* are not yet clear. Heatmap clusters 2 and 3 contain genes that are significantly higher in fetal tenocytes compared to both adult and ESC-tenocytes and include *ADGRL3*, which play a role in cell adhesion, and *LURAP1L*, which is involved in cell migration. *FNDC3A* is involved in extremity development and fin regeneration [90], but its role in mammalian limb development is still to be determined. Other genes in these clusters have no clear role in tendon development.

Interestingly, the expression patterns of many chemokines, cytokines and growth factors, involved in cell migration, were also found to differ between adult and fetal tenocytes. As such, preliminary testing was conducted to investigate the differences in adult and fetal cells’ migratory response to growth factors. Of those tested, TGF-β3, which is heavily involved in the wound repair process [91–93], significantly inhibited fetal tenocyte migration, yet had no effect on adult tenocytes. This observation confirms previous reports in skin and oral mucosa fibroblasts [85, 94, 95]. Interestingly, TGF-β3 has been implicated as a “critical traffic controller” which can selectively halt the migration of certain cell types during skin wound repair to ensure proper wound closure [96]. Further investigations to underpin the potential mechanism behind this differing response to TGF-β3 and the effect of TGF-β3 on ESC-tenocytes are warranted.

One limitation of our migration studies is that investigations were conducted on conventional plastic substratum. It has previously been reported that cytokine and growth factor actions are substratum-dependant [95, 97, 98] which highlights the need to develop assay systems which more closely resemble the in vivo environment. Our results highlight this point, with only ten genes being found to be significantly different between the 2D-cultured adult and fetal tenocytes, compared to the 542 genes which were DE in 3D culture. This supports previous studies showing that distinct gene expression profiles between cell types isolated from different stages [99], and different tissues [23], are better preserved in 3D culture. What was more surprising was that adult and fetal tenocytes appeared to modulate gene expression differently when exposed to a 3D culture environment, with very few DE genes overlapping when comparing the two. Unfortunately, we were unable to compare these results to the native tissue; therefore, further research is necessary to confirm whether this change in expression under 3D culture mimics that of their true in vivo counterpart.

## Conclusion

In summary, this study demonstrates that there are significant differences in tenocyte populations cultured in 3D at different developmental stages. ESC-tenocytes were transcriptomically closer to fetal than adult tenocytes. We were also able to add further evidence as to the benefits of 3D culture as opposed to conventional monolayer passaging. Future studies to optimise this 3D model to allow study of the wound healing environment in adult and fetal cells is likely to prove fruitful, and our transcriptomic data may help to identify key modulators involved in the scaring process as well as highlight if ESC-tenocytes will indeed prove a useful therapeutic.

## Supplementary information

**Additional file 1: Table S1.** Primer Sequences used for qPCR. **Table S2.** Primary and secondary antibodies used for immunocytochemistry. **Table S3.** Gene Analytics pathway analysis of differentially expressed genes in 3D cultured equine tenocytes. The Gene Analytics pathway analysis tool was used to determine the top 10 pathways which are upregulated for each pairwise comparison of DE genes in adult, fetal and ESC-tenocytes based on entity score. **Table S4.** GO analysis of DE genes between adult and fetal tenocytes cultured in 2D versus 3D. Summary of the significantly enriched gene ontology (GO) biological process terms. The panther GO-slim statistical overrepresentation test tool was used to determine over representation of defined GO classification for the 183 genes that were commonly DE between adult and fetal cells in 2D versus 3D culture. **Table S5.** Gene Analytics pathway analysis of DE genes in 2D versus 3D cultured tenocytes. **(A)** The GeneAnalytics pathway analysis tool used to determine the top pathways which are upregulated in monolayer cultures of fetal and adult tenocytes compared to 3D fetal and adult cultures respectively. **(B)** The GeneAnalytics pathway analysis tool used to determine the top pathways which are upregulated in 3D cultures of fetal and adult tenocytes compared to monolayer fetal and adult cultures respectively.

**Additional file 2: Figure S1.** Box and whisker plots of COL1A2, SCX, THBS4 and TNMD expression over serial passaging. **(A)***COL1A2* expression at P0 is significantly higher than at P6 and P7. **(B)***SCX* expression at P0 is significantly higher than at P4 through to P10. However, after P4 there is no significant change in *SCX* expression with further passage. **(C)***THBS4* expression at P0 is significantly higher than at P1 through to P10. However, after P4 there is no significant change in *THBS4* expression with further passage. **(D)***TNMD* expression at P0 is significantly higher than at P1 through to P10. However, after P1 there is no significant change in *TNMD* expression with further passage. Error bars represent the st.dev of 3 biological adult tenocyte lines. Statistical significance tested using linear regression analysis in R (v.3.5.2), with an asterisk (*) denoting statistical significance using P0 as the intercept. **Figure S2.** Additional Haematoxylin and Eosin (H&E) and Picro Sirius Red Staining Images. Hematoxylin and Eosin (H&E) and Picro Sirius Red staining of the remaining two biological lines of fetal, adult and ESC-derived 3D constructs. All cell types show similar collagen fiber alignment and collagen content within the constructs after 14 days of culture. Scale bar for H&E = 0.5 mm. Scale bar for Picro Sirius Red = 250 μm. Under light microscopy all collagen fibers are red following Picro Sirius Red staining, under polarized microscopy collagen type I fibers have yellow-orange birefringence and collagen type III fibers have green birefringence. **Figure S3.** Heatmap of top 100, 200 and 500 DE genes in adult and fetal tenocytes. Differential gene expression analysis for top 100, 200 and 500 genes DE in adult and fetal tenocytes. Dendrograms are based on Pearson correlation, with red and blue colours representing up and down regulated genes respectively. Samples with intermediate expression are represented in yellow. Groups are visualized in columns, with the coloured bar above the heatmap indicating the grouping variable. Genes are represented in individual rows, with patterns of up and down regulated genes being classified into clusters.

**Additional file 3.** Gene level summaries.

## Data Availability

The RNA-sequencing datasets supporting the conclusions of this article are available in the National Centre for Biotechnology Information Gene Expression Omnibus repository (NCBI GEO, www.ncbi.nlm.nih.gov/geo) [accession number GSE145029]. The differential expression analysis and normalised counts datasets supporting the conclusions of this article are included within in Additional file 3. The remaining data used and/or analysed during the current study are available from the corresponding author upon reasonable request.

## Abbreviations

2D: Two-dimensional
3D: Three-dimensional
AT: Achilles tendon
DE: Differential expression
DMEM: Dulbecco’s modified Eagles’ medium
ECM: Extracellular matrix
ESCs: Embryonic stem cells
FBS: Fetal bovine serum
FDR: False discovery rate
GO: Gene ontology
H&E: Haematoxylin and eosin
LIF: Leukaemia inhibitory factor
Log2FC: Log 2-fold change
LQ: l-glutamine
MSCs: Mesenchymal stromal cells
P: Passage
P/S: Penicillin/streptomycin
p-adj: adjusted *p* value
PCA: Principle component analysis
qPCR: Quantitative polymerase chain reaction
RNA-seq: RNA sequencing
SDFT: Superficial digital flexor tendon
TGF: Transforming growth factor
