# Supplementary material for: Genome-wide transcriptome analysis reveals equine embryonic stem cell-derived tenocytes resemble fetal, not adult tenocytes
Source: Stem Cell Res Ther. 2020 May 19;11:184. doi: 10.1186/s13287-020-01692-w (PMC7238619; doi:10.1186/s13287-020-01692-w)
Supplement: Supplementary file 1 — Additional file 1: Table S1. Primer Sequences used for qPCR. Table S2. Primary and secondary antibodies used for immunocytochemistry. Table S3. Gene Analytics pathway analysis of differentially expressed genes in 3D cultured equine tenocytes. The Gene Analytics pathway analysis tool was used to determine the top 10 pathways which are upregulated for each pairwise comparison of DE genes in adult, fetal and ESC-tenocytes based on entity score. Table S4. GO analysis of DE genes between adult and fetal tenocytes cultured in 2D versus 3D. Summary of the significantly enriched gene ontology (GO) biological process terms. The panther GO-slim statistical overrepresentation test tool was used to determine over representation of defined GO classification for the 183 genes that were commonly DE between adult and fetal cells in 2D versus 3D culture. Table S5. Gene Analytics pathway analysis of DE genes in 2D versus 3D cultured tenocytes. (A) The GeneAnalytics pathway analysis tool used to determine the top pathways which are upregulated in monolayer cultures of fetal and adult tenocytes compared to 3D fetal and adult cultures respectively. (B) The GeneAnalytics pathway analysis tool used to determine the top pathways which are upregulated in 3D cultures of fetal and adult tenocytes compared to monolayer fetal and adult cultures respectively. [file 13287_2020_1692_MOESM1_ESM.docx]

| Gene | Forward Primer | Reverse Primer |
| --- | --- | --- |
| 18s rRNA | CCCAGTGAGAATGCCCTCTA | TGGCTGAGCAAGGTGTTATG |
| CDK1 | CTGGGCAGTTCATGGATTCT | CAATCCCTTGTAGGATTTGG |
| COL1A2 | GTGCCTAGCAACATGCCAAT | GTCCTCTATCTCCGGTTGGG |
| COL14A1 | CTTCATGTTCTGCCTACGGG | CGTCTTGGACAGGGGTGAAT |
| IL1R1 | GAAGCGCATAAAGGGCACTA | CGTGGGCCTGATTTCATCTA |
| IL1R2 | TCTGGCACCTACATCTGCAC | CAGGGCAGCTTCTGTCTTCT |
| IL1RN | GCCTGTGTCAAGTCTGGTGA | CCTCCTTGTTCTTGCTCAGG |
| MKX | AAGGCAAAGGAACCATTCGG | TTAGCTGTCACCCTTATTGGAT |
| MMP13 | GCCACTTTGTGCTTCCTGAT | CGCATTTGTCTGGTGTTTTG |
| SCX | CCCAAACAGATCTGCACCTT | ATCCGCCTCTAACTCCGAAT |
| SMAD3 | ATCCCCAAATCCGATGTCCC | GCGCTGGTTCAGCTCATAGT |
| SOX11 | GTTCATGGTGTGGTCCAAGA | GCTGTCCTTCAGCATTTTCC |
| THBS4 | GGGAAATGGGGTTACCTGTT | CGGGTAGCAGGGATGATATT |
| TNMD | GTCCCTCAAGTGAAGGTGGA | CCTCGACGGCAGTAAATACAA |

**Table S1.** **Primer sequences used for qPCR**

**Table S2. Primary and secondary antibodies used for immunocytochemistry**

| Primary Antibodies | | | |
| --- | --- | --- | --- |
| Antibody Target | **Species** | **Dilution** | **Company** |
| Actin, muscle | mouse | 1:200 | Dako (M0635) |
| BMP7 | rabbit | 1:100 | Abcam (ab56023) |
| Collagen type 1 | mouse | 1:100 | Abcam (90395) |
| COMP | rabbit | 1:500 | Kindly provided by Professor Roger Smith, Royal Veterinary College, UK |
| IGF1 | goat | 1:50 | Abcam (ab106836) |
| LOXL4 | rabbit | 1:100 | Biorbyt (orb100094) |
| PDGFB | rabbit | 1:50 | Abcam (ab181341) |
| RUNX2 | rabbit | 1:50 | Santa Cruz (sc10758) |
| SCX | rabbit | 1:100 | Abcam (ab58655) |
| SMAD2/3 (C-8) | mouse | 1:100 | Santa Cruz (sc133098) |
| SPP1 | mouse | 1:50 | Santa Cruz (sc21742) |
| TGF-βR1 | rabbit | 1:100 | Santa Cruz (sc398) |
| TGF-βR2 | rabbit | 1:100 | Santa Cruz (sc220) |
| THBS4 | rabbit | 1:100 | Santa Cruz (sc7657-R) |
| TNC | rabbit | 1:100 | Abcam (ab108930) |
| Secondary Antibodies | | | |
| Antibody Target | **Fluorescence** | **Dilution** | **Company** |
| Anti-mouse IgG | Alexafluor 594 | 1:200 | Thermo Fisher (A11005) |
| Anti-rabbit IgG | Alexafluor 594 | 1:200 | Thermo Fisher (A11012) |
| Anti-mouse IgG H&L | FITC | 1:200 | Abcam (ab7064) |
| Anti-goat IgG H&L | FITC | 1:200 | Abcam (ab7121) |

**Table S3. Gene Analytics pathway analysis of differentially expressed genes in 3D cultured equine tenocytes.**

| **GeneAnalytics Pathway Analysis** | **Entity Score** | **Number of Genes in Superpath** | **Number of Genes Matched** |
| --- | --- | --- | --- |
| **Adult vs Fetal** | | | |
| ERK Signalling | 39.55 | 1180 | 60 |
| Akt Signalling | 23.78 | 682 | 35 |
| Signalling by GPCR | 22.52 | 2599 | 84 |
| PAK Pathway | 22.14 | 683 | 34 |
| TGF-Beta Pathway | 22.02 | 653 | 33 |
| RET Signalling | 21.48 | 972 | 42 |
| PI3K-Akt Signalling Pathway | 20.48 | 524 | 28 |
| Degradation of the Extracellular Matrix | 20 | 297 | 20 |
| Microglia Activation During Neuroinflammation | 19.82 | 71 | 10 |
| Cytokine Signalling in Immune System | 18.74 | 760 | 34 |
| **Adult vs ESC-Derived** | | | |
| ERK Signalling | 166.1 | 1180 | 257 |
| Degradation of the Extracellular Matrix | 67.19 | 297 | 103 |
| Phospholipase-C Pathway | 48.42 | 500 | 128 |
| Akt Signalling | 43.21 | 682 | 154 |
| Integrin Pathway | 41.08 | 568 | 133 |
| Cell Junction Organization | 39.2 | 134 | 51 |
| PAK Pathway | 39.18 | 683 | 150 |
| Apoptotic Pathways in Synovial Fibroblasts | 36.38 | 727 | 154 |
| PI3K-Akt Signalling Pathway | 32.34 | 524 | 117 |
| Activation of CAMP-Dependent PKA | 32.26 | 630 | 134 |
| **Fetal vs ESC-Derived** | | | |
| ERK Signalling | 166.1 | 1180 | 239 |
| Degradation of the Extracellular Matrix | 64.66 | 97 | 97 |
| Phospholipase-C Pathway | 53 | 500 | 125 |
| Akt Signalling | 46.29 | 682 | 149 |
| PAK Pathway | 46.12 | 683 | 149 |
| Integrin Pathway | 39.78 | 568 | 125 |
| CREB Pathway | 35.7 | 529 | 115 |
| Activation of CAMP-Dependent PKA | 34.25 | 630 | 129 |
| PI3K-Akt Signalling Pathway | 33.43 | 524 | 112 |
| Angiogenesis (CST) | 31.53 | 89 | 89 |

The Gene Analytics pathway analysis tool was used to determine the top 10 pathways which are upregulated for each pairwise comparison of DE genes in adult, fetal and ESC-tenocytes based on entity score.

**Table S4. GO analysis of DE genes between adult and fetal tenocytes cultured in 2D versus 3D.**

| **PANTHER GO-Slim Overrepresented Biological Process** | **REFLIST COUNT (14707)** | **INPUT COUNT** | **INPUT (expected)** | **INPUT (Fold Enrichment)** | **INPUT (Raw P-value)** | **INPUT (FDR)** | **Genes** |
| --- | --- | --- | --- | --- | --- | --- | --- |
| **2D vs 3D** | | | | | | |  |
| Cell Adhesion (GO:0007155) | 191 | 13 | 2.55 | 5.09 | 2.95E-06 | 2.57E-03 | CDH23, ITGB4, RASL12, NOV, CDH3, VCAM1, SPON1, CELSR1, CDH1, WISP2, CD80, SVEP1, ITGB8 |
| Cell Surface Receptor Signalling (GO:0007166) | 350 | 16 | 4.68 | 3.42 | 2.66E-05 | 1.54E-02 | ENSECAG00000000954, GRIK5, ENSECAG00000006218, ITGB4, RASL12, IGFBP5, TASPAN11, PTCHD1, CELSR1, SEMA6D, FGF7, CD80, TGFA, PDGFA, LCP2, ITGB8 |

Summary of the significantly enriched gene ontology (GO) biological process terms. The panther GO-slim statistical overrepresentation test tool was used to determine over representation of defined GO classification for the 183 genes that were commonly DE between adult and fetal cells in 2D versus 3D culture.

**Table S5. Gene Analytics pathway analysis of DE genes in 2D versus 3D cultured tenocytes. (A)**

| **Source** | **SuperPath** | **Score** | **Genes Involved** | **Source** | **SuperPath** | **Score** | **Genes Involved** |
| --- | --- | --- | --- | --- | --- | --- | --- |
| **Upregulated in Adult Monolayer Culture** | Cardiac Conduction | 22.84 | KCNJ4, ACTA2, ACTG2, ATP12A, DYSF, LMOD1, KCNK15, NPR1, RYR1, TPM1, TBX5, RYR2, DES | **Upregulated in Fetal Monolayer Culture** | Diseases of Glycosylation | 22.04 | ADAMTS14, ADAMTS6, ADAMTSL3, CSPG5, GPC3, OGN, MUC1, THBS2, THBS1, SDC2 |
|  | Vascular Smooth Muscle Contraction | 18.99 | KCNJ4, ACTA2, ADRA1A, ADM, ADORA1, ACTG2, ADORA2A, CALCB, CALCA, EDN1, NPR1, RYR1, RYR2 |  | Phospholipase-C Pathway | 20.44 | FGF19, EPYC, ACTA2, ACTA1, ADCY5, COL11A1, FGF21, ELN, FGF5, OGN, PPP1R14A, CCN2, COL5A2, ITGA7, MYL9, ITGA11, ITGA8, COL12A1, PLA2G2A, PLA2G5, TGFA, GDF6 |
|  | Smooth Muscle Contraction | 15.84 | ACTA2, ACTG2, DYSF, LMOD1, TPM1 |  | Degradation of the Extracellular Matrix | 18.94 | ADAMTS14, COL11A1, ELN, FBLN5, MMP17, COL5A2, MFAP4, ITGA7, ITGA11, ITGA8, NCAM1, COL12A1, LOX, THBS1, SDC2, SERPINE1 |
|  | Muscular Dystrophies and Dystrophin Glycoprotein Complex | 15.3 | ACTA2, ACTA1, ACTG2, DTNA |  | ERK Signalling | 17.99 | FGF19, EPYC, EPHB3, ACTA2, LIMS2, ACTA1, ADCY5, COL11A1, IL16, FGF21, EPOR, ELN, ATF5, CACNA1H, FGF5, CDH23, MAP3K5, OGN, CAMK2D, PPP1R14A, MUC1, CDC42EP3, MAP3K8, CCN2, COL5A2, ITGA7, MYL9, ITGA11, ITGA8, COL12A1, PRKAA2, TGFA, GDF6, GLI1, DES |
|  | Peptide Ligand Binding Receptors | 13.63 | GABRA5, ADRA1A, ADM, HTR1B, ADORA1, ADORA2A, HRH2, CALCB, CALCA, EDN1, CGA, NPFFR2, TRH, F2RL2, PTGDR, TBXA2R, BDKRB1 |  | Integrin Pathway | 17.51 | EPYC, ACTA2, ACTA1, ADCY5, COL11A1, AOC3, ELN, CACNA1H, MAP3K5, CAMK2D, RASSF5, COL5A2, MMP23B, ITGA7, MYL9, ITGA11, ITGA8, COL12A1, MMP28, THBS1, SDC2, SERPINE1 |
|  | Myometrial Relaxation and Contraction Pathways | 12.95 | ACTA2, ADRA1A, ACTA1, ADM, IGFBP5, IGFBP3, CALCA, RYR1, RYR2 |  | Dilated Cardiomyopathy | 16.95 | ADCY5, EDN1, ITGA7, ITGA11, ITGA8, PRKAA2, TPM1, RYR2, DES |
|  | Cytoskeleton Remodelling | 11.93 | ACTA2, ACTA1, ACTG2, REPS2 |  | Vascular Smooth Muscle Contraction | 16.95 | KCNJ4, ACTA2, ADCY5, ADM, AGTR1, EDN1, CAMK2D, PPP1R14A, NPR1, MYL9, SLC25A4, PRKAA2, PLA2G2A, PLA2G5, RYR2 |
|  | Degradation of the Extracellular Matrix | 11.83 | ADAMTS14, COL11A1, LAMA4, ELN, P3H2, ITGB3, MFAP4, NCAM1, THBS1 |  | Striated Muscle Contraction | 15.79 | ACTA2, ACTA1, MYL9, MYOM1, TPM1, DES |
|  | G Alpha Signalling Events | 11.73 | ADM, ADORA2A, HRH2, CALCB, CALCA, CGA, PTGDR |  | Cardiac Conduction | 14.18 | KCNH2, KCNJ4, ACTA2, DYSF, LMOD1, KCNK15, CAMK2D, NPR1, MYL9, TPM1, RYR2, DES |
|  | EPHA Forward Signalling | 11.67 | ACTA2, ACTA1, EPHA4, LYN |  | Amino Acid Synthesis and Interconversion | 14.1 | ASNS, GLS2, GPT2, PSAT1, PHGDH |

**(B)**

| **Source** | **SuperPath** | **Score** | **Genes Involved** | **Source** | **SuperPath** | **Score** | **Genes Involved** |
| --- | --- | --- | --- | --- | --- | --- | --- |
| **Upregulated in Adult 3D Culture** | ERK Signalling | 24.9 | GAS1, FZD8, ACAN, COL13A1, CXCL14, ITGA10, FGF7, BIRC3, IGF1, ITGB4, FGF18, BMPR1B, FZD10, LTBP4, PDGFRA, CCL2, HAPLN2, TNFRSF21, SPON1, CACNA1G, PLCE1, PTN, MYH15, CDH1, CLEC11A, CCL1, CCL7, STAT4, NCF2, CDH3, COL6A3, MAP3K9, TCF7, PLCB1, TDGF1, RARG, TNFRSF25, ITGB8 | **Upregulated in Fetal 3D Culture** | Cytokine Immune System | 47.14 | EREG, IL18, ITGAX, IL36G, DUSP2, FGF7, BIRC3, DDX58, B2M, IRF7, GBP5, IRF1, IFIT2, GBP6, IL15, FGF1, IL11, IRF8, CCL5, FGF2, CD80, PDGFRA, CCL2, LIF, TNFRSF4, SOCS2, CD40, RASA2, IL1R1, NOS2, OASL, RET, IFIT3, SHC3, STAT1, NRG1, MMP9, TRIM2, VCAM1, RSAD2, XAF1, TNFRSF18, PSMB10, MMP1, PTGS2, TEC, PSMB8, TNFSF8, OAS2, TNFSF11, HGF, S1PR1, IL1RL2, ISG20, UBA7 |
|  | Degradation of the Extracellular Matrix | 20.22 | CTSL, ACAN, COL13A1, CTSK, ITGA10, JAM2, ITGB4, LTBP4, MMP16, CDH1, MMP9, COL6A3, LRP4, MMP13, VCAM1, ITGB8 |  | PAK Pathway | 39.74 | ACVRL1, DLL1, CXCL14, IL18, FGF7, ANGPT2, FGF12, BMP7, BMP6, BMP2, BMPR1B, IL15, FGF1, IL11, CCL5, FGF2, CD80, PDGFRA, NECTIN3, PRKAR2B, CCL2, LIF, TNFRSF4, SPON1, CD40, IL1R1, SEMA3A, MYH15, CDH1, PLPP3, RND3, CCL1, CCL7, NCF2, PTPRE, NRG1, PLA2G7, PTPRO, PTPRK, PTPRN, PLA2G4A, TNFRSF18, TCF7, NTRK1, TNFSF10, TNFSF8, TNFSF11, HGF |
|  | Integrin Pathway | 19.09 | ACAN, COL13A1, CXCL14, GSN, ITGA10, JAM2, ITGB4, CCL2, HAPLN2, SPON1, CACNA1G, MYH15, CCL1, CCL7, STAT4, NCF2, MMP9, COL6A3, MMP13, VCAM1, PLCB1, ITGB8 |  | NF-kappaB Signalling | 38.68 | LCP2, DDX58, B2M, IRF7, IRF1, IRF8, CCL5, CD74, CD274, CCL2, TNFRSF4, SOCS2, CD82, PRDM1, PTGS1, STAT4, NR4A1, STAT1, PECAM1, VCAM1, RSAD2, TNFRSF18, TBX21, TAP2, PTGS2, TEC, PSMB8, TNFSF11, REL, UBD, TLR3, CARD11 |
|  | Akt Signalling | 18.31 | GAS1, CXCL14, ITGA10, FGF7, LCP2, IGF1, ITGB4, FGF18, BMPR1B, LTBP4, PDGFRA, CCL2, TNFRSF21, SPON1, PLCE1, PTN, CLEC11A, CCL1, CCL7, STAT4, PLCB1, TDGF1, TNFRSF25, ITGB8 |  | Akt Signalling | 36.16 | ACVRL1, CXCL14, IL18, ITGAX, ITGA2, FGF7, ANGPT2, FGF12, LCP2, BMP7, GNG11, BMP6, ITGB4, BMP2, BMPR1B, IL15, FGF1, IL11, CCL5, ETV1, FGF2, CD80, PDGFRA, CCL2, LIF, TNFRSF4, SPON1, CD40, IL1R1, SEMA3A, PLPP3, RND3, CCL1, CCL7, STAT4, STAT1, NRG1, TNFRSF18, TEC, NTRK1, TNFSF10, TNFSF8, TNFSF11, HGF, ITGB8, TLR3 |
|  | PAK Pathway | 18.27 | GAS1, CXCL14, FGF7, IGF1, FGF18, BMPR1B, LTBP4, PDGFRA, CCL2, TNFRSF21, SPON1, PLCE1, PTN, MYH15, CDH1, CLEC11A, CCL1, CCL7, NCF2, PTPRD, TCF7, PLCB1, TDGF1, TNFRSF25 |  | ERK Signalling | 34.8 | ACVRL1, COL13A1, CXCL14, IL18, ITGAX, ITGA2, FGF7, ANGPT2, FGF12, ARHGEF3, BIRC3, BMP7, GNG11, BMP6, ITGB4, MCF2L, BMP2, BMPR1B, IL15, FGF1, IL11, CCL5, FZD10, FGF2, CD80, PDGFRA, PRKAR2B, CCL2, HAPLN2, LIF, TNFRSF4, SPON1, CD40, CACNA1G, IL1R1, NOS2, SEMA3A, MYH15, CDH1, COL24A1, PLPP3, RND3, CDH8, CCL1, CCL7, STAT4, CDH17, NCF2, STAT1, NRG1, CDH3, NET1, SPOCK3, TNFRSF18, PTGS2, TCF7, NTRK1, TNFSF10, TNFSF8, TNFSF11, HGF, ITGB8, WNT5A |
|  | Phospholipase-C Pathway | 18.19 | GAS1, ACAN, COL13A1, ITGA10, FGF7, LCP2, IGF1, ITGB4, FGF18, LTBP4, PDGFRA, HAPLN2, SPON1, PLCE1, PTN, CLEC11A, COL6A3, PLCB1, TDGF1, ITGB8 |  | Innate Immune System | 32.83 | CTSH, EREG, ADAM8, IL18, ITGAX, IL36G, CTSC, AP1S2, DUSP2, FGF7, LCP2, BIRC3, DDX58, B2M, IRF7, BPIFB4, GBP5, IRF1, IFIT2, GBP6, ATP8B4, IL15, FGF1, C5AR2, IL11, C2, C3AR1, IRF8, CAMK4, C1S, CCL5, CXADR, EEA1, CD74, FGF2, CD200, CD80, CD274, PDGFRA, IER3, PRKAR2B, CCL2, LIF, TNFRSF4, SOCS2, CD40, RASA2, IL1R1, NOS2, SNAP25, CDH1, OASL, RET, PDE1C, IFIT3, SHC3, SERPINB10, MMP25, NLRP3, NR4A1, NCF2, STAT1, NRG1, MMP9, PECAM1, SLC27A2, TRIM2, VCAM1, RSAD2, XAF1, TNFRSF18, PSMB10, TAP2, MMP1, PTGS2, TEC, PLAUR, PRKG1, PSMB8, TNFSF8, OAS2, TNFSF11, HERC6, HGF, REL, S1PR1, IL1RL2, ISG20, UBA7, TLR3, CARD11 |
|  | Apoptotic Pathways in Synovial Fibroblasts | 16.78 | GAS1, GSN, ITGA10, FGF7, LCP2, IGF1, ITGB4, FGF18, LTBP4, PDGFRA, SPON1, CACNA1G, PLCE1, PTN, CLEC11A, CRABP2, NCF2, SERPINF1, MMP9, RBP5, TDGF1, PERP, RARG, ITGB8 |  | TGF-Beta Pathway | 31.09 | IL18, ITGAX, ITGA2, FGF7, ANGPT2, FGF12, ARHGEF3, BMP7, GNG11, BMP6, ITGB4, MCF2L, BMP2, IL15, FGF1, IL11, CCL5, FGF2, CD80, PDGFRA, CCL2, SOCS2, SPON1, IL1R1, SEMA3A, CDH1, PLPP3, CCL1, CCL7, STAT4, STAT1, NRG1, NET1, TNFRSF18, PSMB10, TCF7, NTRK1, TNFSF10, PSMB8, HGF, ITGB8, UBD |
|  | ECM-Receptor Interactions | 16.16 | COL13A1, ITGA10, JAM2, ITGB4, CDH1, COL6A3, VCAM1, ITGB8, THBS4 |  | Pathways in Cancer | 27.11 | HHIP, DLL1, ITGA2, EDNRA, HEY2, FGF7, BIRC3, AXIN2, GNG11, BDKRB2, BMP2, IL15, FGF1, FZD10, FGF2, EDNRB, PDGFRA, NOS2, CDH1, RET, EPAS1, STAT4, STAT1, LPAR6, MMP9, NKX3-1, WNT16, MMP1, PTGS2, TCF7, NTRK1, HGF, WNT11, WNT7B, WNT5A |
|  | TGF-Beta Pathway | 15.96 | GAS1, ITGA10, FGF7, IGF1, ITGB4, FGF18, LTBP4, PDGFRA, CCL2, SOCS2, SPON1, PTN, CDH1, CLEC11A, CCL1, CCL7, STAT4, MAP3K9, TCF7, TDGF1, TNFRSF25, ITGB8 |  | Immune Response IFN Alpha/beta Signalling Pathway | 25.75 | IL18, IRF7, IRF1, IFIT2, IL15, IRF8, OASL, IFIT3, STAT1, RSAD2, XAF1, PSMB8, OAS2, ISG20 |
|  | Nanog in Mammalian ESC Pluripotency | 13.38 | GAS1, FZD8, FGF7, IGF1, FGF18, FZD10, LTBP4, PDGFRA, SPON1, PLCE1, PTN, CDH1, CLEC11A, CDH3, TCF7, PLCB1, TDGF1, WNT11 |  | Degradation of the Extracellular Matrix | 24.31 | ADAMTS1, COL13A1, ADAM8, ITGAX, ITGA2, JAM2, BMP7, ITGB4, F11R, BMP2, FGF2, NID1, MMP14, CDH1, COL24A1, MMP25, MMP9, PECAM1, SPOCK3, MMP13, VCAM1, MMP1, TLL1, ITGB8 |

**(A)** The GeneAnalytics pathway analysis tool used to determine the top pathways which are upregulated in monolayer cultures of fetal and adult tenocytes compared to 3D fetal and adult cultures respectively. **(B)** The GeneAnalytics pathway analysis tool used to determine the top pathways which are upregulated in 3D cultures of fetal and adult tenocytes compared to monolayer fetal and adult cultures respectively.
