# Supplementary material for: Genome-wide transcriptome analysis reveals equine embryonic stem cell-derived tenocytes resemble fetal, not adult tenocytes
Source: Stem Cell Res Ther. 2020 May 19;11:184. doi: 10.1186/s13287-020-01692-w (PMC7238619; doi:10.1186/s13287-020-01692-w)
Supplement: Supplementary file 2 — Additional file 2: Figure S1. Box and whisker plots of COL1A2, SCX, THBS4 and TNMD expression over serial passaging. (A)COL1A2 expression at P0 is significantly higher than at P6 and P7. (B)SCX expression at P0 is significantly higher than at P4 through to P10. However, after P4 there is no significant change in SCX expression with further passage. (C)THBS4 expression at P0 is significantly higher than at P1 through to P10. However, after P4 there is no significant change in THBS4 expression with further passage. (D)TNMD expression at P0 is significantly higher than at P1 through to P10. However, after P1 there is no significant change in TNMD expression with further passage. Error bars represent the st.dev of 3 biological adult tenocyte lines. Statistical significance tested using linear regression analysis in R (v.3.5.2), with an asterisk (*) denoting statistical significance using P0 as the intercept. Figure S2. Additional Haematoxylin and Eosin (H&E) and Picro Sirius Red Staining Images. Hematoxylin and Eosin (H&E) and Picro Sirius Red staining of the remaining two biological lines of fetal, adult and ESC-derived 3D constructs. All cell types show similar collagen fiber alignment and collagen content within the constructs after 14 days of culture. Scale bar for H&E = 0.5 mm. Scale bar for Picro Sirius Red = 250 μm. Under light microscopy all collagen fibers are red following Picro Sirius Red staining, under polarized microscopy collagen type I fibers have yellow-orange birefringence and collagen type III fibers have green birefringence. Figure S3. Heatmap of top 100, 200 and 500 DE genes in adult and fetal tenocytes. Differential gene expression analysis for top 100, 200 and 500 genes DE in adult and fetal tenocytes. Dendrograms are based on Pearson correlation, with red and blue colours representing up and down regulated genes respectively. Samples with intermediate expression are represented in yellow. Groups are visualized in columns, with the coloured bar ab [file 13287_2020_1692_MOESM2_ESM.docx]

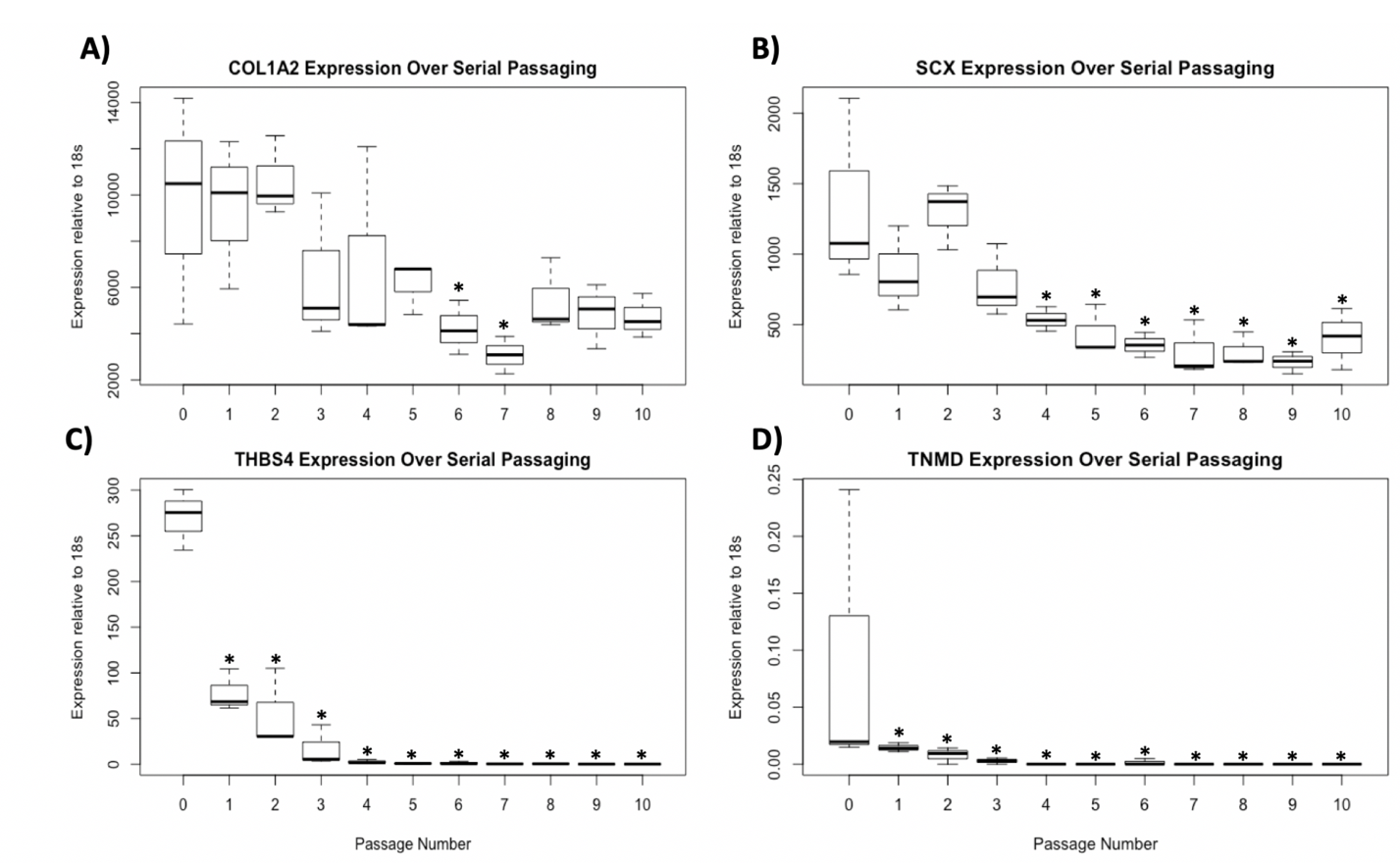


**Fig.S1*.* Box and whisker plots of COL1A2, SCX, THBS4 and TNMD expression over serial passaging. (A)** *COL1A2* expression at P0 is significantly higher than at P6 and P7. **(B)** *SCX* expression at P0 is significantly higher than at P4 through to P10. However, after P4 there is no significant change in *SCX* expression with further passage. **(C)** *THBS4* expression at P0 is significantly higher than at P1 through to P10. However, after P4 there is no significant change in *THBS4* expression with further passage. **(D)** *TNMD* expression at P0 is significantly higher than at P1 through to P10. However, after P1 there is no significant change in *TNMD* expression with further passage. Error bars represent the st.dev of 3 biological adult tenocyte lines. Statistical significance tested using linear regression analysis in R (v.3.5.2), with an asterisk (*) denoting statistical significance using P0 as the intercept.


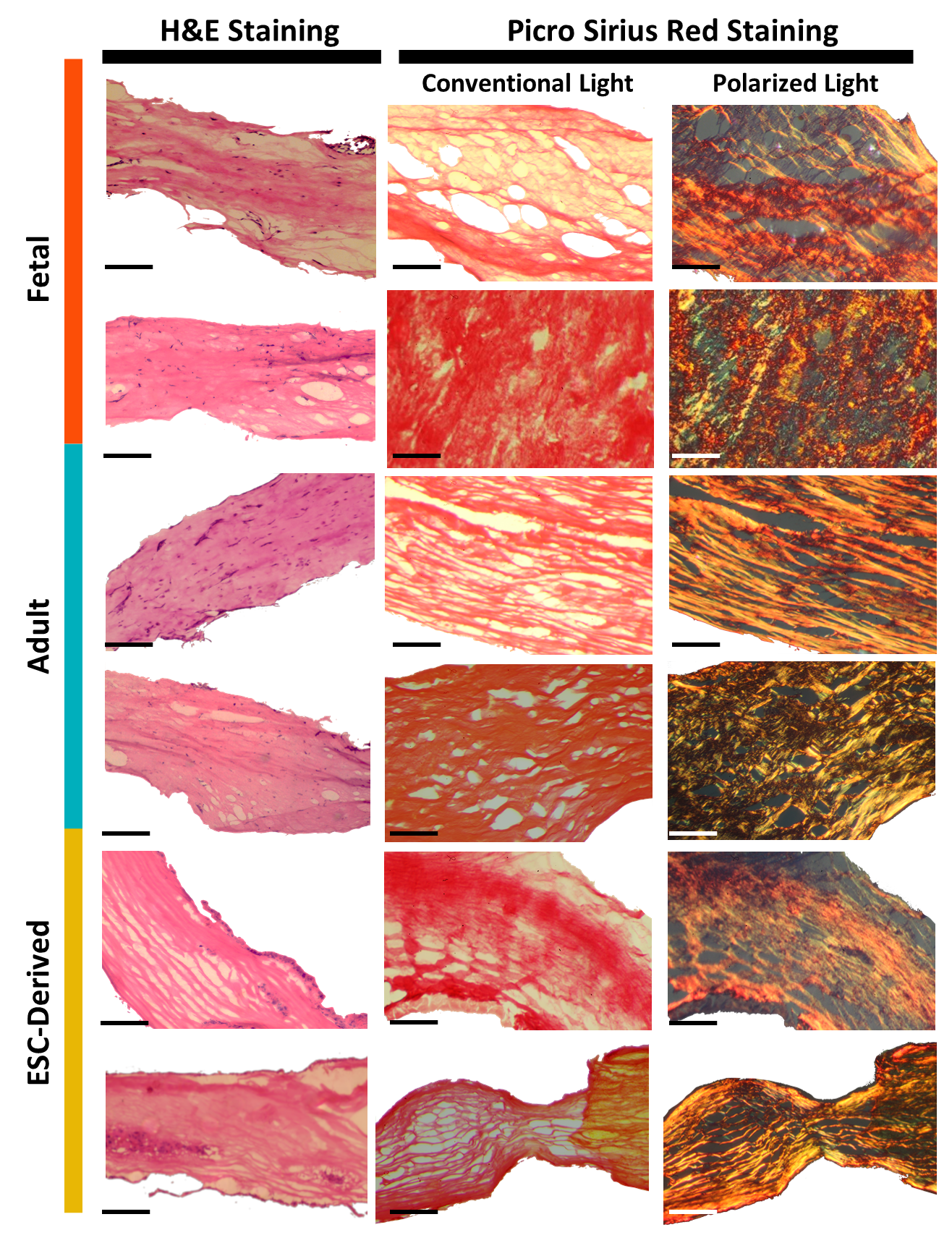


**Fig.S2*.* Additional Haematoxylin and Eosin (H&E) and Picro Sirius Red Staining Images.** Hematoxylin and Eosin (H&E) and Picro Sirius Red staining of the remaining two biological lines of fetal, adult and ESC-derived 3D constructs. All cell types show similar collagen fiber alignment and collagen content within the constructs after 14 days of culture. Scale bar for H&E = 0.5 mm. Scale bar for Picro Sirius Red = 250 µm. Under light microscopy all collagen fibers are red following Picro Sirius Red staining, under polarized microscopy collagen type I fibers have yellow-orange birefringence and collagen type III fibers have green birefringence.


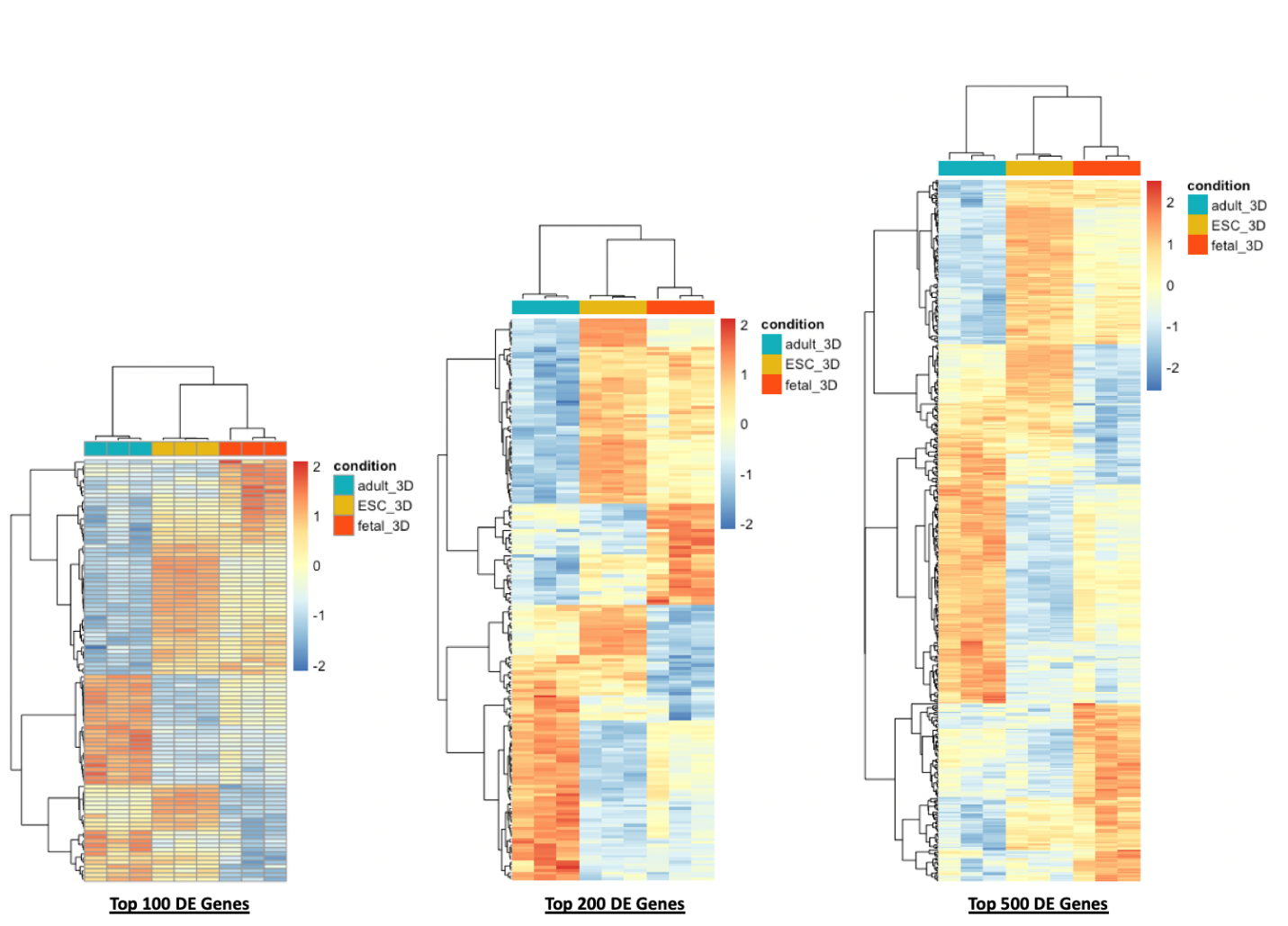


**Fig.S3*.* Heatmap of top 100, 200 and 500 DE genes in adult and fetal tenocytes.** Differential gene expression analysis for top 100, 200 and 500 genes DE in adult and fetal tenocytes. Dendrograms are based on Pearson correlation, with red and blue colours representing up and down regulated genes respectively. Samples with intermediate expression are represented in yellow. Groups are visualized in columns, with the coloured bar above the heatmap indicating the grouping variable. Genes are represented in individual rows, with patterns of up and down regulated genes being classified into clusters.
